# Volumetric‐modulated arc therapy versus intensity‐modulated radiotherapy for large volume retroperitoneal sarcomas: A comparative analysis of dosimetric and treatment delivery parameters

**DOI:** 10.1002/acm2.12230

**Published:** 2017-11-27

**Authors:** Amandeep S. Taggar, Darren Graham, Elizabeth Kurien, James L. Gräfe

**Affiliations:** ^1^ Department of Radiation Oncology University of Toronto Toronto ON Canada; ^2^ Odette Cancer Centre Sunnybrook Health Sciences Centre Toronto ON Canada; ^3^ Division of Radiation Oncology Tom Baker Cancer Centre Calgary AB Canada; ^4^ Cumming School of Medicine Department of Oncology University of Calgary Calgary AB Canada; ^5^ Department of Physics Ryerson University Toronto ON Canada

**Keywords:** dosimetry, intensity‐modulated radiotherapy (IMRT), retroperitoneal sarcoma, volumetric‐modulated arc therapy (VMAT)

## Abstract

**Purpose:**

To compare dosimetric and treatment delivery parameter differences between volumetric‐modulated arc radiotherapy (VMAT) and intensity‐modulated radiotherapy (IMRT) for large volume retroperitoneal sarcomas (RPS).

**Materials and Methods:**

Both VMAT and IMRT planning were performed on CT datasets of 10 patients with RPS who had been previously treated with preoperative radiotherapy. Plans were optimized to deliver ≥95% dose to the PTV and were evaluated for conformity and homogeneity. Dose to the organs at risk (OARs) (kidney, liver, spinal cord, and bowel space), unspecified tissue, and dose evaluation volumes (DEVs) at 1, 2, and 5 cm from PTV were calculated and compared. Monitor units (MUs) and treatment delivery times were recorded and compared between the two techniques. The deliverability of the large volume RPS VMAT plans was verified by portal dosimetry on a Truebeam™ linac.

**Results:**

VMAT and IMRT plans were equivalent for PTV coverage and homogeneity (*P* > 0.05); however, VMAT plans had slightly better conformity index, CI (*P* < 0.001). Doses to the OARs were not significantly different between VMAT and IMRT plans (*P* > 0.05). Mean doses to the unspecified tissue as well as at 1, 2, and 5 cm DEVs were lower with VMAT compared with IMRT,* P* = 0.04 and *P* < 0.01, respectively. MUs and average beam‐on times were both significantly lower in the VMAT vs IMRT plans, *P* < 0.001 and *P* = 0.001, respectively. All VMAT plans passed portal dosimetry delivery verification with an average gamma passing rate of 99.6 ± 0.4%.

**Conclusions:**

VMAT planning for large volume RPS improved CI, and achieved comparable OAR sparing, as compared with IMRT. As treatment delivery time was lower, the use of VMAT for RPS may translate into improved treatment delivery efficiency.

## 1 INTRODUCTION

Retroperitoneal sarcomas (RPS) are rare tumors, comprising approximately 15% of sarcomas. Surgery is the mainstay of treatment for patients with resectable disease; 5‐year overall survival is 50%–60%.^1^ The predominant pattern of failure after surgery is loco‐regional.^2^, ^3^, ^4^ Although prospective randomized trials evaluating the role of radiotherapy (RT) for RPS are lacking, multiple retrospective institutional series suggest that RT improves local control and disease‐free survival^5^, ^6^, ^7^, ^8^, ^9^ vs surgery alone. An expert panel on the treatment of RPS recommended the use of preoperative RT as compared with postoperative RT for several reasons: (a) the dose required preoperatively is lower, (b) reduction in the volume of organs at risk (OARs) receiving RT, and (c) more accurate target volume definition.^10^^.^

Treatment planning and delivery has vastly improved in the last two decades. Inverse planning systems such as intensity‐modulated RT (IMRT) have an advantage of improving target coverage while sparing normal organs over 3D conformal RT.^11^, ^12^, ^13^ RPS are typically large in size and are adjacent to multiple dose‐limiting normal organs, making RPS a challenging nonuniform subgroup of tumors where improved treatment planning and treatment delivery would be highly desirable. IMRT is the currently recommended technique for treatment of RPS by expert panel consensus.^10^ Unfortunately, delivery of IMRT plans on average can take 20–30 min.^14^ Longer treatment times have an impact on the workflow throughput of a treatment unit and uncertainty of target and OAR dose calculations due to intra‐fraction motion.^15^ One potential solution to overcome these issues is to use volumetric‐modulated arc therapy (VMAT).^16^ Llacer‐Moscardo et al. reported on feasibility of VMAT in seven preoperative and three postoperative RPS cases and implied it is superior than IMRT‐based plans.^17^ However, a direct comparison study of VMAT versus IMRT treatment planning within the same cohort of patients has not been reported.

The objectives of this study were to directly compare dosimetric and treatment delivery parameter differences between VMAT and sliding window IMRT (swIMRT) in patients treated with preoperative RT for RPS.

## 2 METHODS

After receiving study approval for this retrospective planning study from our local institutional research ethics board, 10 patients with RPS who were treated with preoperative RT in 2012–2013 at our institution were identified. Planning CT datasets were retrieved from the Eclipse™ (Varian Medical Systems, Palo Alto, CA, USA) treatment planning system (TPS), and each case was re‐planned with both VMAT and swIMRT. In order to ensure consistency over all plans, both the IMRT and VMAT plans were generated by the same planner and the stopping criterion was based on the dosimetric goals listed in the next section.

### 2.A Plan generation, dosimetric considerations, and conformity index

The previously defined gross tumor volumes (GTV), clinical target volumes (CTV), and planning target volumes (PTV) were utilized. Organs at risk (OARs) — liver, kidney, spinal cord, and bowel space — were contoured either for actual treatment or for the purposes of this study if they were not done previously. New plans were generated using analytical anisotropic algorithm (AAA) in Varian Eclipse™ v. 11.0.31, using a prescribed dose to the PTV of 45 Gy in 25 fractions. IMRT plans were generated using 4–6 co‐planar beams. Beam angles were customized based on size and location of the PTV. Dose volume optimizer (DVO) v. 11.0.31 was used to optimize the IMRT plans. VMAT plans were generated using 2–4 partial arcs. The number of arcs and arc start and stop angles were customized, based on size and location of the PTV. Progressive resolution optimizer (PRO) v. 11.0.31 was used to optimize the plans. All IMRT and VMAT plans were optimized and calculated using 6‐MV photons to deliver greater than 95% of the prescription dose to 95% of PTV (D95), while respecting OAR dose constraints based on QUANTEC.^18^^.^

Conformity was assessed using the van't Reit conformity index (CI).^19^ The CI is defined as less than or equal to 1; if CI value is closer to 1, it is considered to be more conformal. Homogeneity was assessed using ICRU83 definition of homogeneity index, defined as $\frac{D2\%-D98\%}{D50\%}$. The dose to the unspecified tissue (all of tissue that is not contoured as a target or an OAR) was also recorded. Dose evaluation volumes (DEVs) at 1, 2, and 5 cm (D1, D2, and D5 cm) expansions from the PTV were created. These structures were trimmed to the body contour in instances where they extended outside the body. Mean doses in DEVs were recorded to estimate dose fall‐off. Monitor units (MU) were obtained and treatment delivery times were measured using mock runs of each plan on a treatment unit.

### 2.B Plan deliverability and quality assurance (QA)

The deliverability of the VMAT plans was verified using portal dosimetry with an electronic portal imaging device (EPID) mounted on a Varian Truebeam™ linear accelerator (linac) (Varian Medical Systems, Palo Alto, CA, USA) using a 43 × 43 cm^2^ aSi Digital Megavolt Imager (DMI). This newly developed large area detector allowed for delivery verification of the large field VMAT plans. The plans were assessed based on the gamma criteria^20^ of 3%/3 mm with a clinical passing threshold of 95% of points using Varian's Portal Dosimetry software.

### 2.C Statistical considerations

All dosimetric comparisons were performed using nonparametric statistical models in Microsoft Excel (Microsoft Corp, Seattle, WA, USA).

## 3 RESULTS

Table 1 summarizes the patient and tumor characteristics. Mean tumor volume was 2433 cm^3^ (standard deviation, SD = 3471 cm^3^), and mean PTV was 3311 cm^3^ (SD = 3287 cm^3^).

**Table 1:** Patient, tumor, and treatment characteristics

Patient characteristics	
Age, median (range)	62.0 yr (32.2–76.2 yr)
Male, n	4
Female, n	6
Treatment	
Preoperative RT, n	10
Radical surgery, n	9
Palliative surgery, n	1
Histology, n	
Liposarcoma	6
Spindle cell sarcoma	2
Leiomyosarcoma	1
Pleomorphic sarcoma	1
Tumor volume, mean (SD)	2433 cm^3^ (3471 cm^3^)
PTV, mean (SD)	3311 cm^3^ (3287 cm^3^)

PTV, planning target volume; RT, radiotherapy; SD, standard deviation.

Table 2 summarizes dosimetric parameters for all 10 patients. On average, 94.2% of the PTV was covered by 95% of the dose with VMAT plans as compared with 92.5% with swIMRT (*P* = 0.5). VMAT plans had a better CI, 0.88 (SD = 0.03) compared with swIMRT plans 0.85 (SD = 0.03) (*P* < 0.0001), but both plans had similar homogeneity within the PTV, 0.068 and 0.066 (*P* > 0.05), respectively. Doses to the OARs were not significantly different for VMAT and swIMRT plans. Mean doses to the unspecified tissue and DEVs (D1, D2, and D5 cm) were significantly lower for VMAT plans compared with swIMRT plans, *P* = 0.04 and *P* < 0.01, respectively. VMAT plans required 490 MUs, 53% lower than swIMRT plans, *P* < 0.0001.

**Table 2:** Dosimetric comparisons of target coverage and OARs between VMAT and swIMRT

	VMAT	swIMRT	*P*‐value
Number of arcs/beam angles, median (range)	2.5 (2–4)	5 (4–6)	<0.001
PTV coverage (D95), mean (range)	94.2% (88.8–97.7)	92.5% (80.0–100.0)	0.5
CI, mean (range)	0.88 (0.83–0.93)	0.85 (0.80–0.91)	<0.001
Uninvolved contralateral kidney mean dose (Gy), (range)	7.0 (2.2–10.1)	7.3 (2.3–12.8)	0.8
Liver mean dose (Gy), mean (range)	14.9 (2.7–23.1)	15.0 (2.8–22.4)	0.6
Spinal cord max dose (Gy), mean (range)	26.9 (12.0–39.7)	29.6 (16.0–45.1)	0.1
Bowel space (D195 cm^3^) (Gy) (range)	38.7 (30.2–46.0)	40.5 (33.5–47.4)	0.1
Unspecified tissue mean dose (Gy), mean (range)	15.3 (11.3–21.7)	15.9 (12.3–22.0)	0.04
DEVs (Gy), mean (range)			
1 cm from PTV	39.9 (36.7–41.8)	40.9 (39.0–42.3)	<0.001
2 cm from PTV	33.2 (30.4–36.8)	34.6 (31.7–36.9)	<0.001
5 cm from PTV	21.7 (18.4–26.3)	22.4 (18.8–26.1)	0.009
Monitor units, mean (range)	490 (367–725)	1042 (610–1570)	<0.001
Treatment delivery time (min), mean (SD)	1.75 (0.66)	7.24 (1.18)	<0.001

CI, conformity index; D195 cm^3^, dose to 195 cm^3^ of bowel space; DEV, dose evaluation volume; Gy, SI unit of dose; OAR, organs at risk; PTV, planning target volume; SD, standard deviation; swIMRT, sliding window intensity‐modulated radiotherapy; VMAT, volumetric‐modulated arc therapy.

The measured average beam‐on time, as determined by delivering the individual VMAT plans in QA mode on the Truebeam™ linac, was 1.75 min (SD = 0.66 min), which was significantly lower when compared with actual beam‐on time for swIMRT plans as delivered during treatment, 7.24 min (SD = 1.18 min) (*P* < 0.001). All VMAT plans passed the portal dosimetry delivery verification at greater than 98.5% of points passing the gamma criterion of 3%/3 mm. The average gamma passing rate was 99.6 ± 0.4% for all VMAT plans. These large field sarcoma plans are, therefore, indeed deliverable at clinical tolerances. The mean gamma value for all VMAT fields was 0.22 ± 0.04. This is significantly less than the threshold value of 1 and much less than 0.5, which demonstrates that there were no systematic issues in absolute treatment delivery.^21^^.^

## 4 DISCUSSION

Preoperative RT is recommended for RPS to reduce local recurrence.^4^, ^10^ Improvement in target dose delivery and reduction of dose to OARs with IMRT compared with 3D‐CRT has been established.^22^, ^23^, ^24^ VMAT is an improved and more efficient method of delivering IMRT as demonstrated for other tumor sites.^25^, ^26^, ^27^, ^28^, ^29^, ^30^, ^31^, ^32^, ^33^ A single feasibility study investigating the use of VMAT for treatment of RPS is reported^17^; however, they did not perform a direct comparison of VMAT to IMRT as it has been done for other tumor sites. Therefore, it is important to conduct and report dosimetric comparison studies of VMAT and IMRT in RPS, such that evidence can guide adoption of this technique in clinical practice.

Similar to published literature,^29^, ^34^, ^35^, ^36^ D95 in this study was comparable between VMAT and IMRT plans (*P* > 0.5); however, CI was statistically improved in favor of VMAT (*P* < 0.001). The target volumes in this study, however, were significantly larger than reported by others. Our portal dosimetry measurements demonstrate that the VMAT beams are deliverable for these large volumes (PTV volumes ranging from 415 to 10194 cc). The homogeneity index ($\frac{D2\%-D98\%}{D50\%})$ was 0.068 and 0.066 for VMAT and IMRT, respectively. This is comparable to earlier reported RPS studies (D5%–D95%).^17^^.^

Reduction of dose to uninvolved critical organs close to the target is an important factor when considering adoption of a new technique. This is especially important in the case of RPS, where large tumors often lie very close to critical structures, such as kidneys. Therefore, any potential dose reduction especially to the uninvolved contralateral kidney may confer therapeutic gain. Jansen et al. have shown the incidence of late kidney injury up to 52% when V20 (volume of kidney receiving 20 Gy) and if mean kidney dose were higher than 66% of prescribed 45 Gy in 25 fractions.^37^ The mean doses to uninvolved contralateral kidney with VMAT and IMRT in this study were significantly lower compared with Jansen et al. and are comparable to those reported by Llacer‐Moscardo et al.^17^ Moreover, we observed a further reduction of mean dose by 4.1% with VMAT compared with swIMRT. Similar reductions in dose were also noticed for other OARs (Table 2); these were not statistically significant, likely due to a small sample size.

Low dose bath of radiation especially from IMRT has been implicated in a potential increase in secondary malignancies.^38^, ^39^ Therefore, naturally it is assumed that risk of secondary malignancies would be even higher with arc therapy, where the low dose bath of radiation is splayed over even a larger area. One way to estimate the low dose bath is to measure dose to unspecified tissue outside the PTV and OARs. This is the first study to report mean dose to unspecified tissue outside the target and OARs, and it was significantly lower with VMAT compared with swIMRT (*P* = 0.04). Furthermore, we report dose fall‐off from PTV by generating spherical volumes around the PTV. This method allows us to estimate intermediate‐ to low‐dose gradient. This is typically performed in SBRT plans, where dose at 2 cm is used to optimize the plan to generate sharper dose fall‐off and decrease intermediate dose.^40^ In this study, dose fall‐off was measured for three DEVs that we created as dose fall‐off estimating structures from the PTV. The mean dose within all three DEVs was significantly lower for VMAT plans compared with swIMRT plans (*P* < 0.01). This indicates a sharper dose fall‐off with VMAT, and an overall lower intermediate dose around the PTV for these large volume treatment plans.

The biggest advantage of VMAT over IMRT is shorter treatment time.^33^, ^41^, ^42^, ^43^ In this study, there was 53% reduction in average number of MUs and 76% reduction in measured treatment time with VMAT plans compared with swIMRT plans (*P* < 0.001). This is consistent with other published studies that have compared VMAT and IMRT.^29^, ^31^, ^32^, ^34^, ^35^, ^44^, ^45^ The shorter treatment time may translate in improved workflow within a radiation department, as typical IMRT slots are 25–30 min long.^14^ Shorter beam‐on time may result in decreased intra‐fraction motion of the target and OARs during treatment. Zhuang has modeled dose uncertainty in relation to organ motion and field size and concluded that there is higher dose uncertainty with increasing field size and motion amplitude.^15^ The treatment of RPS generally requires large field sizes, and previous literature has documented significant motion of these tumors and adjacent organs, particularly in the upper abdomen.^46^ IMRT plans that require a higher number of MUs and take longer to deliver, therefore, would be more vulnerable to the increased dose uncertainty from intra‐fraction tumor and organ motion. Thus, for RPS patients, VMAT plans that can deliver highly conformal treatment in shorter time may confer a therapeutic advantage, although this hypothesis needs to be assessed in a formal prospective setting. In addition, lower MUs, leading to a shorter beam‐on time, reduce the out of field dose due to a reduction in head leakage.

## 5 LIMITATIONS

We recognize that this retrospective study has inherent biases of patient and treatment selection. Our results, while intriguing, are hypothesis generating. Formal assessment of patient comfort and toxicity was not done in this study. A prospective study comparing the two treatment techniques may confirm our results and allow for assessment of toxicity and patient comfort with each of these techniques.

## 6 CONCLUSIONS

In this study, we compared dosimetry and deliverability of VMAT versus IMRT for large volume targets such as retroperitoneal sarcomas. VMAT is able to generate plans that are comparable in PTV coverage and homogeneity, have a higher conformity, provide comparable or less dose to OARs, but a sharper dose fall‐off. These dosimetric advantages are complemented by the decreased delivery time of VMAT plans and reduced monitor units. This could potentially translate into improved comfort for the patient, reduced intra‐fraction motion, and improved workflow for a busy radiotherapy department.

## CONFLICT OF INTEREST

The authors disclose no conflict of interest.

## References

[1] Mendenhall WM , Zlotecki RA , Hochwald SN , Hemming AW , Grobmyer SR , Cance WG . Retroperitoneal soft tissue sarcoma. Cancer. 2005;104:669–675. 16003776 10.1002/cncr.21264

[2] Porter GA , Baxter NN , Pisters PW . Retroperitoneal sarcoma: a population‐based analysis of epidemiology, surgery, and radiotherapy. Cancer. 2006;106:1610–1616. 16518798 10.1002/cncr.21761

[3] Singer S , Antonescu CR , Riedel E , Brennan MF . Histologic subtype and margin of resection predict pattern of recurrence and survival for retroperitoneal liposarcoma. Ann Surg. 2003;238:358–370; discussion 370‐1. 14501502 10.1097/01.sla.0000086542.11899.38 PMC1422708

[4] Stoeckle E , Coindre JM , Bonvalot S , et al. Prognostic factors in retroperitoneal sarcoma: a multivariate analysis of a series of 165 patients of the French Cancer Center Federation Sarcoma Group. Cancer. 2001;92:359–368. 11466691 10.1002/1097-0142(20010715)92:2<359::aid-cncr1331>3.0.co;2-y

[5] Gilbeau L , Kantor G , Stoeckle E , et al. Surgical resection and radiotherapy for primary retroperitoneal soft tissue sarcoma. Radiother Oncol. 2002;65:137–143. 12464441 10.1016/s0167-8140(02)00283-9

[6] Gronchi A , Lo Vullo S , Fiore M , et al. Aggressive surgical policies in a retrospectively reviewed single‐institution case series of retroperitoneal soft tissue sarcoma patients. J Clin Oncol. 2009;27:24–30. 19047283 10.1200/JCO.2008.17.8871

[7] Lewis JJ , Leung D , Woodruff JM , Brennan MF . Retroperitoneal soft‐tissue sarcoma: analysis of 500 patients treated and followed at a single institution. Ann Surg. 1998;228:355–365. 9742918 10.1097/00000658-199809000-00008 PMC1191491

[8] van Dalen T , Hoekstra HJ , van Geel AN , et al. Locoregional recurrence of retroperitoneal soft tissue sarcoma: second chance of cure for selected patients. Eur J Surg Oncol. 2001;27:564–568. 11520090 10.1053/ejso.2001.1166

[9] Erzen D , Sencar M , Novak J . Retroperitoneal sarcoma: 25 years of experience with aggressive surgical treatment at the Institute of Oncology. Ljubljana. J Surg Oncol. 2005;91:1–9. 15999353 10.1002/jso.20265

[10] Baldini EH , Wang D , Haas RL , et al. Treatment guidelines for preoperative radiation therapy for retroperitoneal sarcoma: preliminary consensus of an international expert panel. Int J Radiat Oncol Biol Phys. 2015;92:602–612. 26068493 10.1016/j.ijrobp.2015.02.013

[11] Chuong MD , Freilich JM , Hoffe SE , et al. Intensity‐modulated radiation therapy vs. 3D conformal radiation therapy for squamous cell carcinoma of the anal canal. Gastrointest Cancer Res. 2013;6:39–45. 23745158 PMC3674462

[12] Freilich J , Hoffe SE , Almhanna K , et al. Comparative outcomes for three‐dimensional conformal versus intensity‐modulated radiation therapy for esophageal cancer. Dis Esophagus. 2015;28:352–357. 24635657 10.1111/dote.12203

[13] Arbea L , Ramos LI , Martinez‐Monge R , Moreno M , Aristu J . Intensity‐modulated radiation therapy (IMRT) vs. 3D conformal radiotherapy (3DCRT) in locally advanced rectal cancer (LARC): dosimetric comparison and clinical implications. Radiat Oncol. 2010;5:17‐717X‐5‐17. 20187944 10.1186/1748-717X-5-17 PMC2845593

[14] Thomas SJ , Vinall A , Poynter A , Routsis D . A multicentre timing study of intensity‐modulated radiotherapy planning and delivery. Clin Oncol (R Coll Radiol). 2010;22:658–665. 20620036 10.1016/j.clon.2010.06.011

[15] Zhuang T . On the effect of intrafraction motion in a single fraction step‐shoot IMRT. Med Phys. 2015;42:4310. 26133628 10.1118/1.4922687

[16] Otto K . Volumetric modulated arc therapy: IMRT in a single gantry arc. Med Phys. 2008;35:310–317. 18293586 10.1118/1.2818738

[17] Llacer‐Moscardo C , Quenet F , Azria D , Fenoglietto P . Feasibility study of volumetric modulated arc therapy for the treatment of retroperitoneal sarcomas. Radiat Oncol 2010;5:83‐717X‐5‐83. 20854661 10.1186/1748-717X-5-83 PMC2949680

[18] Marks LB , Ten Haken RK , Martel MK . Guest editor's introduction to QUANTEC: a user's guide. Int J Radiat Oncol Biol Phys. 2010;76:S1–S2. 20171501 10.1016/j.ijrobp.2009.08.075

[19] van't RA , Mak AC , Moerland MA , Elders LH , van dZW . A conformation number to quantify the degree of conformality in brachytherapy and external beam irradiation: application to the prostate. Int J Radiat Oncol Biol Phys 1997;37:731–736. 9112473 10.1016/s0360-3016(96)00601-3

[20] Low DA , Harms WB , Mutic S , Purdy JA . A technique for the quantitative evaluation of dose distributions. Med Phys. 1998;25:656–661. 9608475 10.1118/1.598248

[21] Van Esch A , Huyskens DP , Hirschi L , Scheib S , Baltes C . Optimized Varian aSi portal dosimetry: development of datasets for collective use. J Appl Clin Med Phys. 2013;14:82–99. 10.1120/jacmp.v14i6.4286 PMC5714635 24257272

[22] Mock U , Georg D , Bogner J , Auberger T , Potter R . Treatment planning comparison of conventional, 3D conformal, and intensity‐modulated photon (IMRT) and proton therapy for paranasal sinus carcinoma. Int J Radiat Oncol Biol Phys. 2004;58:147–154. 14697432 10.1016/s0360-3016(03)01452-4

[23] Trofimov A , Nguyen PL , Coen JJ , et al. Radiotherapy treatment of early‐stage prostate cancer with IMRT and protons: a treatment planning comparison. Int J Radiat Oncol Biol Phys. 2007;69:444–453. 17513063 10.1016/j.ijrobp.2007.03.018 PMC2695934

[24] Hong L , Hunt M , Chui C , et al. Intensity‐modulated tangential beam irradiation of the intact breast. Int J Radiat Oncol Biol Phys. 1999;44:1155–1164. 10421550 10.1016/s0360-3016(99)00132-7

[25] Popescu CC , Olivotto IA , Beckham WA , et al. Volumetric modulated arc therapy improves dosimetry and reduces treatment time compared to conventional intensity‐modulated radiotherapy for locoregional radiotherapy of left‐sided breast cancer and internal mammary nodes. Int J Radiat Oncol Biol Phys. 2010;76:287–295. 19775832 10.1016/j.ijrobp.2009.05.038

[26] Marnitz S , Wlodarczyk W , Neumann O , et al. Which technique for radiation is most beneficial for patients with locally advanced cervical cancer? intensity modulated proton therapy versus intensity modulated photon treatment, helical tomotherapy and volumetric arc therapy for primary radiation – an intraindividual comparison Radiat Oncol 2015;10:91‐015‐0402‐z. 25896675 10.1186/s13014-015-0402-z PMC4404108

[27] Cozzi L , Dinshaw KA , Shrivastava SK , et al. A treatment planning study comparing volumetric arc modulation with RapidArc and fixed field IMRT for cervix uteri radiotherapy. Radiother Oncol. 2008;89:180–191. 18692929 10.1016/j.radonc.2008.06.013

[28] Clivio A , Fogliata A , Franzetti‐Pellanda A , et al. Volumetric‐modulated arc radiotherapy for carcinomas of the anal canal: a treatment planning comparison with fixed field IMRT. Radiother Oncol. 2009;92:118–124. 19181409 10.1016/j.radonc.2008.12.020

[29] Abbas AS , Moseley D , Kassam Z , Kim SM , Cho C . Volumetric‐modulated arc therapy for the treatment of a large planning target volume in thoracic esophageal cancer. J Appl Clin Med Phys. 2013;14:4269. 23652258 10.1120/jacmp.v14i3.4269 PMC5714417

[30] Liu M , Liu B , Wang H , et al. Dosimetric comparative study of 3 different postoperative radiotherapy techniques (3D‐CRT, IMRT, and RapidArc) for II‐III stage rectal cancer. Medicine (Baltimore). 2015;94:e372. 25569661 10.1097/MD.0000000000000372 PMC4602855

[31] Mellon EA , Javedan K , Strom TJ , et al. A dosimetric comparison of volumetric modulated arc therapy with step‐and‐shoot intensity modulated radiation therapy for prostate cancer. Pract Radiat Oncol. 2015;5:11–15. 25413432 10.1016/j.prro.2014.03.003

[32] Palma D , Vollans E , James K , et al. Volumetric modulated arc therapy for delivery of prostate radiotherapy: comparison with intensity‐modulated radiotherapy and three‐dimensional conformal radiotherapy. Int J Radiat Oncol Biol Phys. 2008;72:996–1001. 18455326 10.1016/j.ijrobp.2008.02.047

[33] Tsai CL , Wu JK , Chao HL , Tsai YC , Cheng JC . Treatment and dosimetric advantages between VMAT, IMRT, and helical tomotherapy in prostate cancer. Med Dosim. 2011;36:264–271. 20634054 10.1016/j.meddos.2010.05.001

[34] Li Z , Zeng J , Wang Z , Zhu H , Wei Y . Dosimetric comparison of intensity modulated and volumetric arc radiation therapy for gastric cancer. Oncol Lett. 2014;8:1427–1434. 25202345 10.3892/ol.2014.2363 PMC4156206

[35] Kataria T , Govardhan HB , Gupta D , et al. Dosimetric comparison between Volumetric Modulated Arc Therapy (VMAT) vs Intensity Modulated Radiation Therapy (IMRT) for radiotherapy of mid esophageal carcinoma. J Cancer Res Ther. 2014;10:871–877. 25579521 10.4103/0973-1482.138217

[36] Myrehaug S , Chan G , Craig T , et al. A treatment planning and acute toxicity comparison of two pelvic nodal volume delineation techniques and delivery comparison of intensity‐modulated radiotherapy versus volumetric modulated arc therapy for hypofractionated high‐risk prostate cancer radiotherapy. Int J Radiat Oncol Biol Phys. 2012;82:e657–e662. 22245189 10.1016/j.ijrobp.2011.09.006

[37] Jansen EP , Saunders MP , Boot H , et al. Prospective study on late renal toxicity following postoperative chemoradiotherapy in gastric cancer. Int J Radiat Oncol Biol Phys. 2007;67:781–785. 17157445 10.1016/j.ijrobp.2006.09.012

[38] Hall EJ . Intensity‐modulated radiation therapy, protons, and the risk of second cancers. Int J Radiat Oncol Biol Phys. 2006;65:1–7. 16618572 10.1016/j.ijrobp.2006.01.027

[39] Weber DC , Johanson S , Peguret N , Cozzi L , Olsen DR . Predicted risk of radiation‐induced cancers after involved field and involved node radiotherapy with or without intensity modulation for early‐stage hodgkin lymphoma in female patients. Int J Radiat Oncol Biol Phys. 2011;81:490–497. 20800383 10.1016/j.ijrobp.2010.05.035

[40] Chang BK , Timmerman RD . Stereotactic body radiation therapy: a comprehensive review. Am J Clin Oncol. 2007;30:637–644. 18091059 10.1097/COC.0b013e3180ca7cb1

[41] Hall WA , Fox TH , Jiang X , et al. Treatment efficiency of volumetric modulated arc therapy in comparison with intensity‐modulated radiotherapy in the treatment of prostate cancer. J Am Coll Radiol. 2013;10:128–134. 23245437 10.1016/j.jacr.2012.06.014

[42] Wolff D , Stieler F , Welzel G , et al. Volumetric modulated arc therapy (VMAT) vs. serial tomotherapy, step‐and‐shoot IMRT and 3D‐conformal RT for treatment of prostate cancer. Radiother Oncol. 2009;93:226–233. 19765846 10.1016/j.radonc.2009.08.011

[43] Rao M , Yang W , Chen F , et al. Comparison of Elekta VMAT with helical tomotherapy and fixed field IMRT: plan quality, delivery efficiency and accuracy. Med Phys. 2010;37:1350–1359. 20384272 10.1118/1.3326965

[44] Shang J , Kong W , Wang YY , Ding Z , Yan G , Zhe H . VMAT planning study in rectal cancer patients. Radiat Oncol 2014;9:219‐014‐0219‐1. 25319073 10.1186/s13014-014-0219-1 PMC4205282

[45] Nguyen K , Cummings D , Lanza VC , et al. A dosimetric comparative study: volumetric modulated arc therapy vs intensity‐modulated radiation therapy in the treatment of nasal cavity carcinomas. Med Dosim. 2013;38:225–232. 23578496 10.1016/j.meddos.2013.01.006

[46] Wong P , Dickie C , Lee D , et al. Spatial and volumetric changes of retroperitoneal sarcomas during pre‐operative radiotherapy. Radiother Oncol. 2014;112:308–313. 25150633 10.1016/j.radonc.2014.08.004

